# Midgut Volvulus Adds a Murine, Neutrophil-Driven Model of Septic Condition to the Experimental Toolbox

**DOI:** 10.3390/cells12030366

**Published:** 2023-01-19

**Authors:** Julia Elrod, Antonia Kiwit, Moritz Lenz, Holger Rohde, Daniela Börnigen, Malik Alawi, Christoph Mohr, Laia Pagerols Raluy, Magdalena Trochimiuk, Jasmin Knopf, Konrad Reinshagen, Martin Herrmann, Michael Boettcher

**Affiliations:** 1 Department of Pediatric Surgery, University Medical Center Mannheim, Heidelberg University, Theodor-Kutzer-Ufer 1-3, 68167 Mannheim, Germany; 2 Department of Pediatric Surgery, University Medical Center Hamburg-Eppendorf, Martinistrasse 52, 20246 Hamburg, Germany; 3 Institute of Medical Microbiology, Virology and Hygiene, University Medical Center Hamburg-Eppendorf, Martinistrasse 52, 20246 Hamburg, Germany; 4 Bioinformatics Core, University Medical Center Hamburg-Eppendorf, Martinistrasse 52, 20246 Hamburg, Germany; 5 Department of Internal Medicine 3-Rheumatology and Immunology, Friedrich-Alexander Universität Erlangen-Nürnberg (FAU) and Universitätsklinikum Erlangen, Ulmenweg 18, 91054 Erlangen, Germany; 6 Deutsches Zentrum für Immuntherapie, Friedrich-Alexander Universität Erlangen-Nürnberg (FAU) and Universitätsklinikum Erlangen, Ulmenweg 18, 91054 Erlangen, Germany

**Keywords:** sepsis, extracellular DNA, sepsis model, volvulus, CLP, LPS

## Abstract

Background: Severe infections that culminate in sepsis are associated with high morbidity and mortality. Despite continuous efforts in basis science and clinical research, evidence based-therapy is mostly limited to basic causal and supportive measures. Adjuvant therapies often remain without clear evidence. The objective of this study was to evaluate the septic volvulus ischemia-reperfusion model in comparison to two already established models and the role of neutrophil extacellular traps (NETs) in this model. Methods: The technique of the murine model of midgut volvulus was optimized and was compared to two established models of murine sepsis, namely cecal ligation and puncture (CLP) and intra-peritoneal (i.p.) injection of lipopolysaccharide (LPS). Results: Midgut volvulus for 15 min caused a comparable mortality (38%) as CLP (55%) and peritoneal LPS injection (25%) at 48 h. While oxidative stress was comparable, levels of circulating free DNA (cfDNA), and splenic/hepatic and pulmonary translocation of bacteria were decreased and increased, respectively at 48 h. DNases were increased compared to the established models. Proteomic analysis revealed an upregulation of systemic Epo, IL-1b, Prdx5, Parp1, Ccl2 and IL-6 at 48 h in comparison to the healthy controls. Discussion and Conclusion: Midgut volvulus is a stable and physiological model for sepsis. Depending on the duration and subsequent tissue damage, it represents a combination of ischemia-reperfusion injury and hyperinflammation.

## 1. Introduction

50 million people worldwide experience sepsis annually with an estimated 11 million deaths; this is equivalent to 20% of all deaths [1,2]. Mortality rates from sepsis only demonstrate a slow decline, which is mainly attributable to improved intensive care management, specific interventions, and campaigns [3,4,5]. In the pediatric population, sepsis is the leading cause of death worldwide, causing 7.5 million deaths of children annually. In the US, 72,000 pediatric patients are hospitalized with sepsis every year with a mortality rate of 25%. This leads to an enormous economic cost of 4.8 billion US$ [6]. Notably, some studies have reported an increasing incidence of pediatric sepsis for certain countries, such as the US. This is related at least in part to the increasing number of high-risk patients with chronic diseases and burdensome conditions such as organ transplants, pediatric cancers and their treatment, or extreme prematurity [7]. Including all acute care hospitalizations for minors under the age of 19, infants have both the highest incidence of sepsis and the highest mortality from sepsis. This is largely due to the extremely high mortality rates among very low birth weight infants (VLBW) [7].

Over the last years, the definition of sepsis has been subject to various refinements [2] Sepsis is currently defined as “organ dysfunction caused by a dysregulated response to infection” [8] emphasizing the pivotal role of the immune system in the etiopathology of this devastating condition [2]. Sepsis begins with a pro-inflammatory response that is soon counteracted by anti-inflammatory processes [9]. A septic pathology arises when the balance of the inflammatory response shifts toward uncontrolled inflammation; massive hyperinflammation with shock, fever, and hypermetabolism develops. Hyperinflammation and coagulopathy are key features of the pathophysiological of sepsis. Disseminated intravascular coagulation (DIC) is driven by impairment of anticoagulatory mechanisms, including antithrombin and the protein C system, by the action of tissue factor, and by the depression of the fibrinolytic system. If sepsis persists, patients enter an immunosuppressive state and eventually die from primary or secondary infections and DIC [9,10]. Neutrophils play a pivotal role in innate immunity in sepsis, inter alia by their release of neutrophil extracellular traps (NETs). NETS have been shown to have both protective and detrimental features in sepsis, the latter by induction of thrombosis and multiple organ failure. Yet many key questions regarding their exact role in the pathophysiology of (pediatric) sepsis remain unanswered [11,12].

In the last few decades there has been some progress in understanding the etiopathology of sepsis. However, the specific therapies currently available to prevent or even alleviate the pathological consequences of sepsis remain limited, despite numerous efforts in clinical and experimental research. Current animal models of sepsis only incompletely reflect human sepsis [13,14,15]. Two commonly employed models are (I) systemic treatment with lipopolysaccharide (LPS), and (II) coecum ligation and puncture (CLP). However, both models have drawbacks: Systemic LPS mainly reflects the cytokine storm. Furthermore, mice are relatively insensitive to LPS. Among other means, the immune response against viable bacteria is missing [16]. In CLP, the polymicrobial flora from the cecum initiates and drives sepsis. This model provides results that have a higher potential for translation. In particular, the cytokine profile more closely resembles those observed in human sepsis. The drawback is that artificial perforation of the otherwise healthy gut is non-physiological and has virtually no correlate in humans [16].

We hypothesize that the volvulus-sepsis model reflects a more comprehensive model of human sepsis. In this model, the intestine is rotated along its axis and rotated back after a defined time. This causes ischemic reperfusion damage with secondary bacterial transmigration. The bacteria migrate through the damaged mucosa into the vascular system and do not enter the abdominal cavity together with stool as in the CLP model. Therefore, there is no artificial perforation of the otherwise healthy intestine. We assume that the inflammatory processes in the volvulus model are more physiological and that the results can therefore be better translated into humans. Importantly, mice are treated with G-CSF, a neutrophil-mobilizing cytokine leading to a significant increase in white blood cell count in order to adapt neutrophil counts in mice to human levels [17,18].

The aims of this study were to technically optimize the volvulus-sepsis model and to compare it with CLP and with peritoneal administration of LPS. Subsequently, the results shall help to understand the etiopathology of sepsis.

## 2. Methods

### 2.1. Study Design

Ethical approval was obtained from the Hamburg State Administration for animal research (TVA 100/17). A total of 76 six to eight-week-old mice were utilized for the experimental model and all environmental parameters within the animal facility complied with the German guide for the care and use of laboratory animals (Tierschutzgesetz). All animals had the same genetic background (C57BL/6).

### 2.2. Animal Procedures

In all mice, anesthesia was induced with 5% isoflurane (Baxter, Unterschleißheim, Germany) and maintained throughout the procedure with 2.5% isoflurane gas delivered through a facemask. Betaisodona was used as preoperative antisepsis. For analgesia, all mice received buprenorphine (Reckitt Benckiser, Mannheim, Germany) 0.1 mg/kg bodyweight subcutaneously, 30 min preoperatively. The mice were randomly divided into four treatment groups (sham, volvulus, CLP, LPS) and the according procedures were performed as described below. To increase standardization, all operations and interventions were performed by the same surgeon. In order to adapt neutrophil counts in mice to human conditions, all mice received 250 µg/kg bodyweight Lenograstim (Granocyte 34, G-CSF Chugai Pharmaceutical, Tokyo, Japan) subcutaneously (s.c.) after the procedure in anesthesia and again after 24 h if not sacrificed at this time point. G-CSF is a neutrophil-mobilizing cytokine [17]. As shown previously, its subcutaneous injection leads to a significant increase in the number of neutrophils in the blood [18]. The additional neutrophil recruitment is intended to compensate for the differences between the human and murine immune systems and thus establishes a more physiological sepsis model [19].

### 2.3. Treatment Groups

Sham: The mice were placed in a dorsal decubitus position while a median laparotomy was performed and the intestine was exposed for 1 min. Finally, the abdomen was closed with simple interrupted sutures using 5 × 0 Prolene. (Total number of animals: *n* = 5)

Volvulus: After performing median laparotomy, the small intestine was exposed and twisted by 720°. The axis of rotation was selected for the intestine to turn livid and signs of congestion became slightly apparent, see supplement VII. The intestine was left in this position for 10, 15, 20 or 30 min, then it was detorsed to its original physiological position. In all animals signs of reperfusion were observed and the abdomen was closed with simple interrupted sutures. (Total number of animals: *n* = 36)

CLP: After performing median laparotomy, the cecum was identified and alloyed with 5 × 0 Vicryl in the midportion. Then two punctures of the distal portion were performed with an 19 G cannula and the abdomen was closed with simple interrupted sutures. (Total number of animals: *n* = 19)

LPS: An i.p. injection of LPS-EB Ultrapure (Escherichia coli O55:B5; Invivogen, San Diego, CA, USA) at a dose of 10 mg/kg bodyweight (1 mg/1 mL LPS) was performed. (Total number of animals: *n* = 16)

After the above-described operations and interventions, anesthesia was discontinued and the animals were housed in the animal facility. For pain control, they henceforth received tramadol (Grünenthal, Aachen, Germany) mixed with their drinking water (1 mg/mL, ad libitum). 24 or 48 h later, relaparotomy was performed using isoflurane as described above and 1 mL of EDTA blood was collected via intracardial puncture. Finally, all animals were euthanatized through cervical dislocation. Subsequently, resection of lung, liver, spleen and small intestine was performed.

### 2.4. Sample Preparation and Evaluation

EDTA blood was centrifuged and the remaining plasma was aliquoted and stored at −80 °C until further use. 100 mg of each organ (lung, liver, spleen, small intestine) was flash frozen using liquid nitrogen and stored at −80 °C, while tissue cylinders (3 mm in diameter) of each organ were preserved for bacterial translocation analysis as described below.

### 2.5. Determination of Bacterial Translocation

The tissue cylinders were lysed using Precellys Lysing Kit (Tissue homogenizing CKMix, Precellys Lysing Kit, Bertin, France), according to the manufacturer’s instruction and under consideration of hygiene standards as to avoid contamination. After dilution of the lysates at 1:5 and 1:100 in PBS, 50 μL were spread out on Columbia agar +5% sheep blood agar (COS), Columbia NaladixicAcid agar (CNA) (Biomerieux, Marcy l’Etoile, France), and Mac Conkey Agar plates (Thermo Fisher Scientific, Waltham, MA, USA) for 48 h at 37 °C, before the number of colony forming units was determined manually. Exemplary mass spectrometric analysis (matrix-assisted laser desorption/ionization (MALDI)) of each macroscopically distinctive unit was then carried out for bacterial identification.

### 2.6. Methods

Circulating free desoxyribonucleic acid (cf-DNA) concentration of cf-DNA was measured in plasma as described previously using a sytoxGreen, fluorescence-based essay [20].

Deoxyribonuclease I (DNase I): Plasma DNase I quantitation, a marker for cell damage, was assessed using the Mouse Deoxyribonuclease I (DNAseI) ELISA kit (MyBioSource, San Diego, CA, USA) according to manufacturer’s instructions.

Glutathione Peroxidase (GPx) Assay: Tissue GPx activity (liver, lung, small intestine), a marker for systemic antioxidant status, was measured using a Glutathione Peroxidase Assay Kit (Cayman, Ann Arbor, MI, USA) according to manufacturer’s instructions. GPx was not reliably measurable in the spleen, most likely because of the different number of erythrocytes that remained in the organ after rinsing it off with PBS.

Malondialdehyde (MDA): Tissue MDA activity (liver, lung, spleen), a marker for lipid peroxidation, was measured using the MDA Assay Kit (Sigma Aldrich, St. Louis, MO, USA) according to manufacturer’s instructions. The MDA activity of the intestine did not differ from the control groups in preliminary tests and was therefore not assessed here.

Proteomic analysis: 25 µL of each frozen plasma sample was shipped to Olink (Uppsala, Sweden) on dry ice. There, the Target 96 Mouse Exploratory panel, based on proximity extension assay (PEA) technology was used to measure 92 proteins. PEA comprises a dual-recognition immune assay, in which two matched antibodies labeled with DNA oligonucleotides bind simultaneously to the target proteins in solution. The proximity of the two antibodies allows hybridization, enabling DNA polymerase dependent extension steps. Further extension and hybridizing steps are followed by PCR amplification and ultimately the proteins are detected on a Fluidigm^®^ Biomark^TM^qPCR instrument [21]. The proteins assayed in the panel encompass a broad range of functions and biological pathways, including amongst others angiogenesis, apoptotic processes, cell motility, cellular metabolic processes and response to stress [22].

Pre-processing and quality control of the data was performed using the Olink NPX Manager software. Samples for which more than half of the proteins were not detectable (below level of detection (LOD)) were removed. Additionally, proteins were removed if they failed to quantify in at least half of the samples. All remaining values below the LOD were substituted by the corresponding protein’s LOD/√2. Finally, one outlier was detected by principal component analysis (PCA) and the corresponding sample was subsequently removed from the analysis.

Each sepsis model (CLP, LSP, Volvulus) was compared to the healthy control (sham). In addition, differential expression of proteins was compared between two time points (24 h and 48 h) for each of the three sepsis models. Differential expression was assessed using the R/limma package. A protein was considered differentially expressed if the corresponding False Discovery Rate (FDR) was below or equal to 0.05 and the absolute log2-Foldchange was above 1.

### 2.7. Statistics

Data analysis was performed using GraphPad Prism 9 (GraphPad, CA, USA) and SPSS Statistics 26 (IBM, NY, USA). G*Power 3.1 was applied for the pre-power study calculation. The power was deducted from previous trials regarding inflammation and NET formation [23]. Differences between groups were calculated using One Way ANOVA with Dunnett’s multiple comparison and Turkey’s test, whenever applicable. Data is presented as mean ± standard deviation (SD). The level of significance was set at 0.05.

## 3. Results

**Figure 1:**
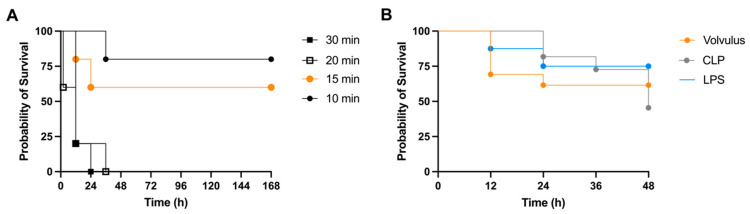
Survival rate in midgut volvulus, cecal ligation and puncture (CLP), and i.p. LPS model. (**A**) Initially, midgut volvulus was performed in a total of 20 mice and left in this position for 10, 15, 20 or 30 minutes before it was relaxed (*n* = 5 per group). Note, the 48 h mortality was 20%, 40%, 100%, and 100%, respectively. (**B**) Survival rate of the CLP (*n* = 11), and the LPS (*n* = 8) sepsis model as compared to midgut volvulus performed for 15 min (*n* = 13) (48 h mortality 38%). CLP model: the midportion of the cecum was alloyed and the distal portion of the cecum was punctured using a 19 G canula (48 h mortality 55%). LPS model: i.p. injection of 10 mg/kg LPS-EB ultrapure (48 h mortality 25%).

At first, a midgut volvulus was induced by twisting the midgut by 720° and keep it in this position for 10, 15, 20 or 30 min, before it was relaxed (*n* = 5 per group). The object of this preliminary experiment was to optimize experimental conditions, e.g., to identify the ideal duration of torsion leading to a tolerable mortality rate. The resulting mortalities were 20% (1/5), 40% (2/5), 100% (5/5) and 100% (5/5) in the 10, 15, 20, and 30 min group after 48 h, respectively (Figure 1A). Next, the midgut volvulus model was repeated in an additional 8 mice for 15 min. The other groups were not repeated, since they lead to either very low or too high mortality rates. Now the overall 48 h mortality was 38% (5/13) in the volvulus model (15 min). The mortality rates in the comparing models were 55% (6/11) in the CLP model and 25% (2/8) in the LPS model (Figure 1B). Importantly, mice from the CLP model continued to die after the 24 h time point.

Note that the survival of volvulus, CLP and i.p. LPS followed similar time kinetics.

Including initial mortality analysis (*n* = 5), the volvulus experiment (t = 15 min) was performed in a total of 13 mice, as to acquire enough tissue and samples for further analysis. The CLP and the i.p. LPS model were performed in less animals to avoid unnecessary animal experiments (*n* = 11 and *n* = 8 respectively).

**Figure 2:**
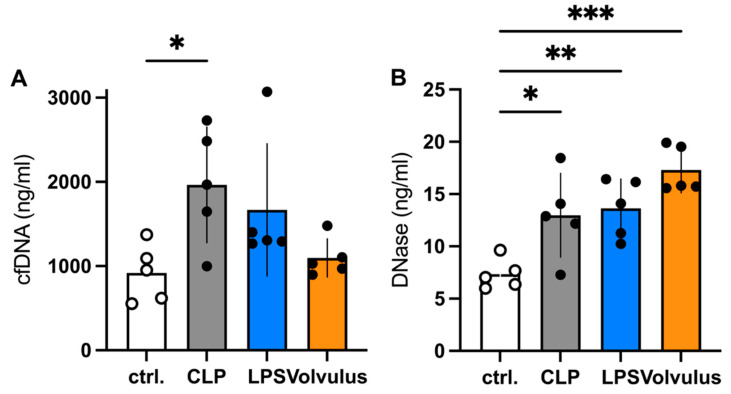
The NET markers cfDNA and DNase I in the blood of mice succumbed to murine models of septic conditions. (**A**) cfDNA and (**B**) DNase plasma levels 48 h after intervention. *n* = 5 per groups. Note, whereas the DNase I was the highest in the volvulus model the cfDNA was close to the controls. Data shown as mean ± SD. * *p* < 0.05, ** *p* < 0.01, *** *p* < 0.001. ANOVA with Dunnett’s correction.

CfDNA, a marker for NET formation or cellular necrosis, was only elevated in the circulation in the CLP group (Figure 2A), whereas plasma DNase levels were increased in all models, when compared with the controls (Figure 2B). The high DNase concentrations are most likely responsible for the low circulating cfDNA levels.

**Figure 3:**
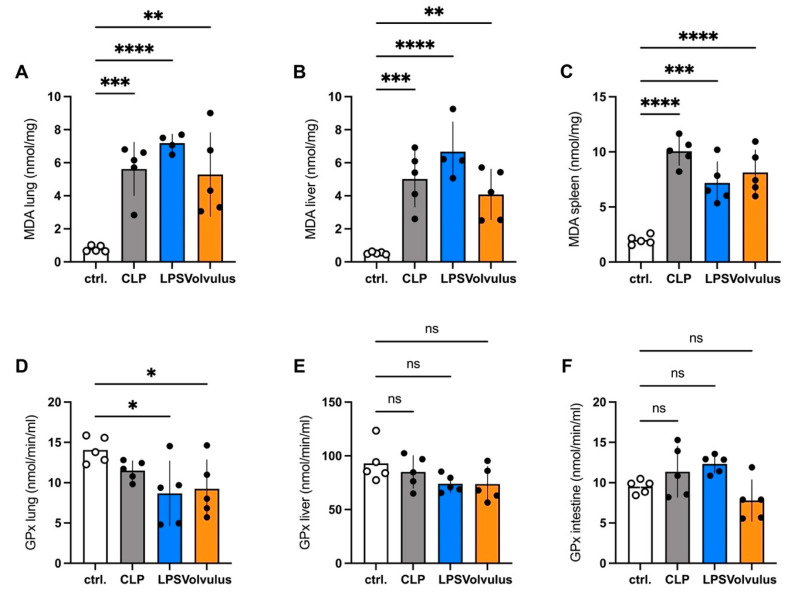
Oxidative stress and antioxidative capacity are increased and decreased in all of the septic conditions, respectively.Oxidative stress resembled by MDA levels (**A**–**C**) is increased and antioxidative capacity resembled by GPx levels (**D**–**F**) is decreased when compared with the controls in most of the organs investigated lung (**A**,**D**), liver (**B**,**E**), spleen (**C**) and intestine (**F**). *n* = 5 in the spleen, *n* = 4 in liver and lung. Data shown as mean ± SD. * *p* < 0.05, ** *p* < 0.01, *** *p* < 0.001, **** *p* < 0.0001, ns = non-significant. ANOVA with Dunnett’s correction.

Oxidative stress in lung, liver and spleen was quantified employing MDA, a marker for lipid oxidation, and GPx, a marker for antioxidative capacity. In the three sepsis models, MDA was increased significantly in lung, liver, and spleen, whereas GPx was significantly decreased only for the LPS and volvulus models in the lung. (Figure 3).

**Figure 4:**
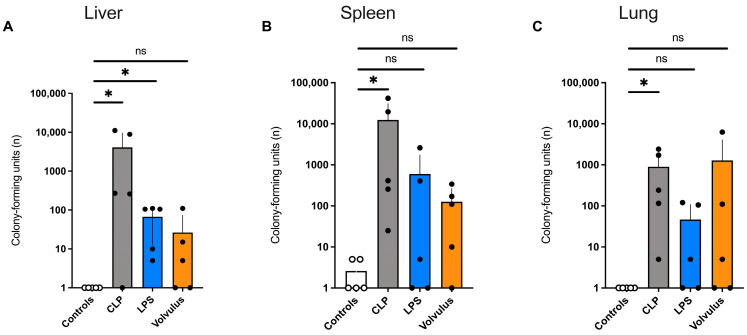
In all three models of septic conditions, bacteria have translocated to liver, spleen and lung. 48 h after intervention CLP, LPS and midgut volvulus led to bacterial translocation into (**A**) liver, (**B**) spleen and (**C**) lung. *n* = 5 in each of the groups. Data shown as mean ± SD. * *p* < 0.05. ns = non-significant. Mann-Whitney-U-Test.

As shown in Figure 4, bacterial translocation is most pronounced in the liver and the spleen of the CLP model. Analyses by MALDI-TOF revealed the 2 most common species to be Enterococcus faecalis and Escherichia coli.

**Figure 5:**
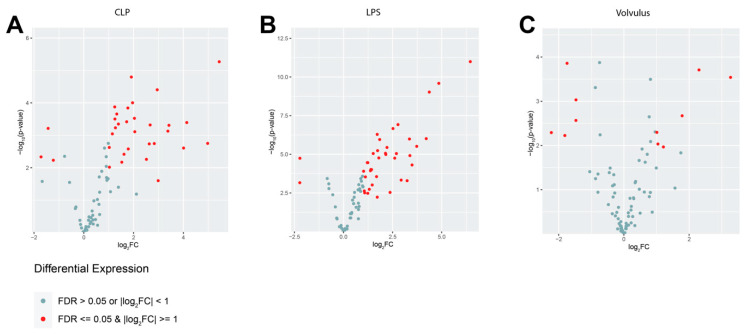
All three models of septic conditions display differential expression of plasma proteins when compared with the controls at 48 h. (**A**) CLP resulted in upregulation of 29 and downregulation of 3 proteins. (**B**) LPS treatment led to upregulation of 37 and downregulation of 2 proteins. (**C**) Midgut volvulus resulted in upregulation of 6 and downregulation of 5 proteins. Expression was defined as significantly different if FDR ≤ 0.05 and |log2FC| ≥ 1 was met. All differentially regulated plasma proteins are listed in supplement I and II.

Several proteins of interest are differentially expressed in each of the three sepsis models compared to healthy controls at 48 h (Figure 5). In the CLP (*n* = 4) model 29 proteins were significantly upregulated, whereas only 3 (Cyr61, IL-23r, Tgfa) were downregulated at 48 h. In the LPS (*n* = 5) model 37 proteins were upregulated, whereas only 2 (Pdgfb, Cyr61) were downregulated at 48 h. In the volvulus (*n* = 3) model, we observed significant upregulation of 6 proteins (Epo, IL-1b, Prdx5, Parp1, Ccl2 and IL-6) and downregulation of 4 proteins (Gcg, IL-23r, Cyr61 and Tgfa) at 48 h.

**Figure 6:**
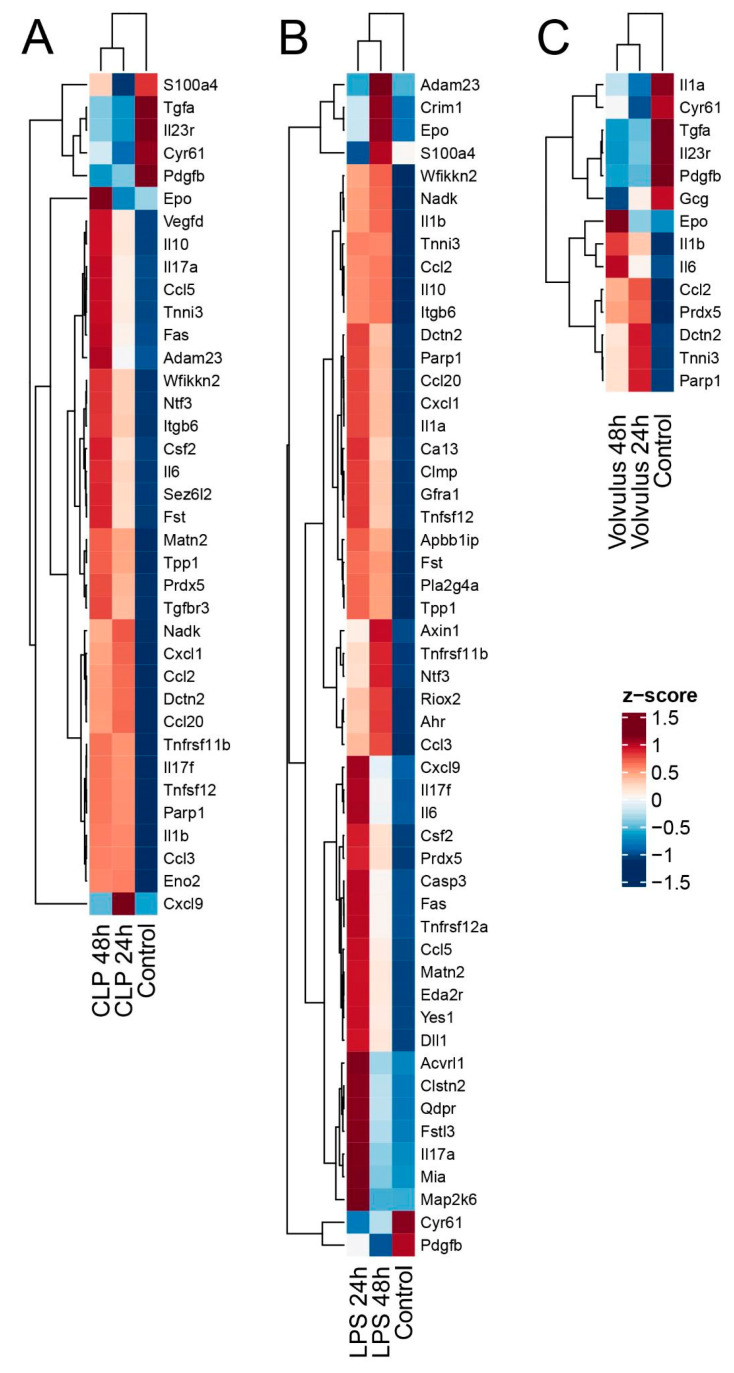
Several plasma proteins are differentially expressed in the three models of septic conditions at 48 h and 24 h in comparison to healthy controls. 48 h and 24 h levels of plasma proteins (**A**) CLP, (**B**) LPS, and (**C**) midgut volvulus are shown together with corresponding controls as heat maps. Z-scores are displayed.

For detailed information regarding differential expression of all proteins at the time points 24 h and 48 h in comparison to healthy controls, see supplement I and II. Next, we analyzed the differences in protein expression at 48 h as compared to 24 h (supplement III). The protein expression was rather stable and changed only very little over time in the CLP model (upregulated: Epo), whereas expression of 13 proteins changed in the LPS model (upregulated: Ccl3, Adam23, S100a4; downregulated: Clstn2, Fstl3, Mia, Matn2, Csf2, Qdpr, IL-6, Ccl5, IL-17a, Cxcl9). No changes were to be observed in the midgut volvulus model. Detailed information is shown in supplement IV. Figure 6 shows a heat map of all differentially expressed proteins for all models at 24 h and 48 h compared to the healthy control.

A detailed look at expression patterns of the pro- and anti-inflammatory proteins was performed as a mean comparison between the groups and is displayed in the supplement V and VI. For this a gene ontology (GO)-based panel search was performed, resulting in 6 pro-inflammatory proteins (TNF, LPl, Ccl3, Pla2g4a, IL-1b, Wisp1) and 3 anti-inflammatory proteins (Ghrl, IL-10, Hgf).

## 4. Discussion

The objective of this study was to evaluate the midgut volvulus model for septic ischemia-reperfusion injury and to compare it with the two most commonly used models of murine sepsis–CLP as well as intraperitoneal injection of LPS. The wider objective of this project is to contribute to the understanding of the pathophysiology of sepsis. Based on our results, the volvulus model conveniently combines two essential features of sepsis, namely hyperinflammation and reperfusion injury. It shows distinct features when compared to the two other models with regards to increased oxidative stress, a reduction in antioxidative capacity, tissue damage, and transmigration of bacteria. It demonstrates a model specific expression of a number of proteins. Overall, our data show that midgut volvulus adds a physiological model to expand the toolbox of research regarding septic conditions and hyperinflammation.

An advantage of the midgut volvulus model is that it is more physiological in comparison to CLP and peritoneal injection of LPS. It represents the highly complex pathophysiological condition that precipitates sepsis. The luminal obstruction and vascular occlusion in midgut volvulus cause mechanical obstruction and bacterial fermentation [24]. These phenomena increase intracolonic pressure, disturb capillary perfusion and clog intramural vessels [25]. With the resulting ischemia, the inflamed mucosa becomes leaky and unable to block bacterial translocation and toxemia. A condition that can culminate in formation of gangrenes and bowel perforation [26]. This differs from CLP where holes are punched into the ligated, yet otherwise healthy intestine in a non-physiological manner. This causes immediate contamination with stool of the abdominal cavity. On the other hand, the animal is initially healthy and the cecal wall is still intact. This mechanism of injury differs from acute perforated appendicitis. The latter is often triggered by an obstruction of the cecal lumen that impedes the flow of mucosal secretions. This increases the intraluminal and intramural pressure, subsequently hinders venous drainage and injures the ischemic mucosa. Certain intraluminal bacteria proliferate and cross the weakened wall of the appendix.

In contrast to the CLP model, perforation and gangrene formation is seen at late stages of volvulus when significant inflammation and necrosis has already occurred [27]. Consequently, a high CFUs count was observed in liver and spleen in mice subjected to CLP. This is likely the consequence from direct bacterial spread per continuitatem due to the perforation of the initially (healthy) cecum. In contrast, in the volvulus model, we observed a reduced number of intrabdominal CFU (liver and spleen) and an increased hematogenous spread of bacteria to the lung. Moreover, we also observed a pertinent number of CFU in liver, spleen and the lung of mice subject to LPS injection. As described previously by others, this finding is most likely to be explained by bacteremia resulting from an increased intestinal barrier permeability and bacterial translocation, which can be found in various systemic and inflammatory diseases and play an important role in the pathogenesis of sepsis induced organ failure [28,29].

Another weakness of murine models for sepsis is the fact that circulating neutrophils are less abundant and display different functions. In humans, blood is rich in neutrophils, while mouse blood has a strong preponderance of lymphocytes [19,30]. In order to adapt neutrophil counts in mice to human levels, we treated the animals with G-CSF, a neutrophil-mobilizing cytokine [17] leading to a significant increase in white blood cell count [18].

As shown in the present study, torsion of the midgut for 15 min was optimal; a shorter duration did not produce sufficient sepsis, reflected by a low mortality, a longer duration was associated with an extremely high mortality. Hence, the 48 h survival rate is similar to the LPS and the CLP model. In the volvulus model, it is possible to adapt the severity of sepsis by varying the degree of rotation. However, this is also a weakness of the model: the accurate reproduction of the degree of ischemia and the consecutive bowel injury requires experimental training to achieve an accurate reproduction of the center of rotation on the axis of the superior mesenteric artery and vein. In our experience, a variation of this pivot point would lead to considerable discrepancies in the result.

In comparison to the other models, the higher DNase and the lower cfDNA levels can be explained by the antagonizing effect of the latter and emphasize the importance of NETs in the volvulus sepsis model. This indicates that the volvulus model is more of a combining sepsis and ischemia-reperfusion injury (I/R) as neutrophils play a crucial role in I/R. They produce reactive oxygen species (ROS), recruit further immune cells and form NETs [31,32,33]. NET formation, a consequence of ROS production, enhances inflammation, intensifies oxidative stress and drives coagulopathy. This ultimately increases damage from I/R [34,35]. The coincidence of I/R with sepsis due to intestinal tissue injury and bacterial transmigration or perforation of the intestinal wall is of interest in certain situations. In others it could also lead to confusion about the specific pathophysiological consequences.

The detailed protein analyses performed, serve as database for further work. In the volvulus model, erythropoietin, IL-1β, mitochondrial peroxiredoxin-5, poly [ADP-ribose] polymerase 1, C-C motif chemokine 2 (CCL2), and IL-6 were upregulated. The most elevated protein was erythropoietin, which is known to be markedly elevated in the initial inflammatory phase of critically ill patients; especially in the context of renal failure [36]. However, erythropoietin also influences the later phase [37]. It is reported to counteract hypotension associated with sepsis by reducing the synthesis of endothelial nitric oxide synthase (eNOS). Furthermore, it shows cardioprotective effects in connection with abdominal sepsis in rats [38].

IL-1β is a proinflammatory protein, which is essential for the pathogenesis of systemic inflammatory responses in the initial phase of sepsis. Upon activation by bacterial ligands macrophages produce IL-1β together with tumor necrosis factor (TNF) and IL-6. IL-1β and TNF activate endothelial cells that, consequently, attract circulating neutrophils to the inflammatory site [39]. Several studies have shown that high levels of IL-6 are associated with increased mortality [40,41]. IL-6 predicts survival of mice undergoing CLP. It can also be used to monitor pharmacological treatments. This qualifies IL-6 as a biomarker of early sepsis [42]. Prdx5 is part of the peroxiredoxin family of antioxidant enzymes. It serves cytoprotective functions during oxidative stress [43]. Parp1 may also contribute to the inflammatory response and the cellular metabolic disorders in sepsis. Antagonism of Parp1 reduces inflammatory responses and mortality [44]. Besides upregulation of a variety of proteins, all three septic conditions also lead to the downregulation of certain proteins. This finding is in line with a previous study assessing differential gene expression in pediatric septic shock, revealing several genes involved in immune response to be downregulated, in addition to a large number of upregulated genes [45].

Here we show that in the volvulus model the proteome only marginally changed between 24 h and 48 h. This is in contrast to the LPS model where significant dynamics have been observed. The fact that no further deaths occurred after 48 h argues for a hit-and-run insult to the bowel. These results are in line with previous findings from a midgut volvulus model in neonatal rats, where no mortality was observed later than 2 days after detorsion [46]. These findings suggest that a plateau in disease activity characterizes the volvulus model, indicating that the volvulus model is a fairly stable model. This is beneficial for certain kinds of analyses. Yet, it is difficult to get a more advanced state of the clinical picture of volvulus, as prolonged duration of torsion resulted in early deaths within few hours after the injury.

Regarding the 3R principle, we argue that the volvulus can help to archive these goals, due to its good reproducibility, standardizability and controllability on the one hand, and its rapid progression and realistic underlying pathophysiology which allow high predictive power, on the other hand. Nevertheless, the use of animal models has to be avoided whenever possible and it should if at all only be used for few specific questions with high clinical relevance for which animal-free models are not yet available and in which the use of the model will most likely yield highly relevant results. Furthermore, research activities should focus on the development of alternatives for animal models, such as Organ-On-A-Chip models, organoids and artificial intelligence-based data analysis and according founds should be provided by the state and universities.

## 5. Conclusions

Midgut volvulus represents a stable model for sepsis that combines I/R and hyperinflammation. However, it requires a certain amount of practice to find the exact pivot point to ensure a reproducible degree of ischemia. The extend of I/R and the inflammatory reaction depends on the duration of the intervention and the consecutive tissue injury. The use of G-CSF is advisable to compensate for the different neutrophil levels in mice versus humans. The advantage of the midgut volvulus model is that it is more physiological compared to the CLP and to intraperitoneal injections of LPS. It represents a highly complex pathophysiological condition leading to I/R and hyperinflammation. By varying the duration and degree of torsion, the model can be easily adjusted to the needs of individual experiments.

## Data Availability

The datasets generated during and/or analysed during the current study are available from the corresponding author on reasonable request.

## Supplementary Materials

The following supporting information can be downloaded at: https://www.mdpi.com/article/10.3390/cells12030366/s1.
