# Supplementary material for: Midgut Volvulus Adds a Murine, Neutrophil-Driven Model of Septic Condition to the Experimental Toolbox
Source: Cells. 2023 Jan 19;12(3):366. doi: 10.3390/cells12030366 (PMC9913099; doi:10.3390/cells12030366)

Supplement I Sepsis models displayed differential expression of plasma proteins when compared with the controls at 24 h.

CLP resulted in upregulation of 25 and downregulation of 5 proteins.

LPS treatment lead to upregulation of 44 and downregulation of 1 protein.

Midgut volvulus resulted in upregulation of 6 and downregulation of 5 proteins.

Expression was considered being significantly different, if FDR ≤ 0.05 and |log2FC| ≥ 1. The differentially regulated plasma proteins are listed in supplemental material II.

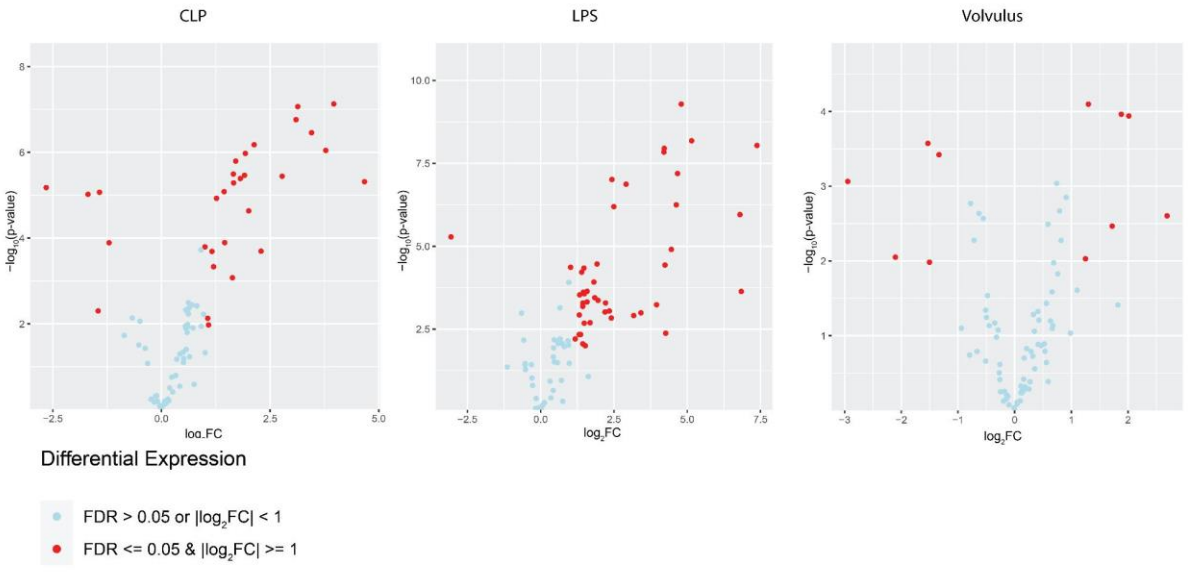

Supplement II: Sepsis models displayed differential expression of plasma proteins when compared with the controls *at* 24h.

CLP 24 h compared to control

Protein	Log2FC	P-Value	FDR	Mean in group CLP24h	Mean in group control
Ccl3	3.96	7.44E-08	0.00000351	6.99	3.02
Tnni3	3.13	8.55E-08	0.00000351	4.64	1.5
Ccl2	3.09	0.000000173	0.00000472	13.18	10.08
IL6	3.45	0.000000349	0.00000715	5.23	1.77
Prdx5	2.13	0.000000665	0.0000109	3.44	1.3
Ccl20	3.78	0.000000901	0.0000123	11.74	7.96
Tnfsf12	1.93	0.00000106	0.0000124	6.41	4.48
Matn2	1.71	0.0000016	0.0000164	6.76	5.05
Tpp1	1.65	0.0000032	0.0000269	8.12	6.46
Ntf3	1.91	0.00000344	0.0000269	3.77	1.86
IL17f	2.78	0.00000361	0.0000269	4.09	1.31
IL10	1.81	0.00000409	0.0000279	3.76	1.94
Cxcl1	4.67	0.00000484	0.0000303	13.48	8.81
Tnfrsf11b	1.66	0.00000517	0.0000303	7.53	5.87
Cyr61	-2.65	0.00000665	0.0000363	3.9	6.55
Dctn2	1.44	0.00000826	0.0000412	2.6	1.16
Tgfa	-1.43	0.00000854	0.0000412	5.46	6.89
IL23r	-1.69	0.00000955	0.0000435	4.24	5.93
Eno2	1.27	0.0000118	0.0000511	4.87	3.6
IL1b	2.01	0.0000233	0.0000955	4.15	2.14
Parp1	1.45	0.000128	0.000481	4.86	3.41
S100a4	-1.21	0.000129	0.000481	3.44	4.65
Wfikkn2	1	0.000161	0.000573	6.27	5.27
Cxcl9	2.29	0.000203	0.000646	7.6	5.31
Sez6l2	1.16	0.000205	0.000646	5.47	4.31
Ccl5	1.2	0.000466	0.00142	2.24	1.04
Csf2	1.63	0.000843	0.00247	2.57	0.94
Pdgfb	-1.46	0.00502	0.0121	6.11	7.57
Nadk	1.06	0.00744	0.0156	7.18	6.12
Fst	1.08	0.0106	0.0208	8.85	7.77

LPS 24 h compared to control:

Protein	Log2FC	P-Value	FDR	Mean in group LPS 24h	Mean in group control
Ccl20	4.8	5.17E-10	4.24E-08	12.76	7.96
Ccl3	5.15	6.6E-09	2.29E-07	8.17	3.02
Ccl5	7.38	9.16E-09	2.29E-07	8.42	1.04
Ccl2	4.22	1.12E-08	2.29E-07	14.3	10.08
Csf2	4.21	1.44E-08	2.37E-07	5.15	0.94
IL10	4.67	6.43E-08	8.79E-07	6.61	1.94
Tnfsf12	2.43	9.74E-08	0.00000114	6.92	4.48
Matn2	2.91	0.000000135	0.00000138	7.96	5.05
IL6	4.63	0.000000563	0.00000513	6.4	1.77
Nadk	2.5	0.000000641	0.00000526	8.62	6.12
Cxcl9	6.8	0.00000111	0.00000829	12.1	5.31
Cyr61	-3.06	0.00000524	0.0000358	3.5	6.55
Cxcl1	4.46	0.0000125	0.0000788	13.27	8.81
Pla2g4a	1.92	0.0000343	0.000201	6.68	4.76
Parp1	4.24	0.0000369	0.000202	7.65	3.41
Tpp1	1.02	0.0000432	0.000221	7.48	6.46
Clstn2	1.47	0.0000457	0.000221	3.8	2.33
Riox2	1.4	0.0000605	0.000275	3.47	2.07
Dctn2	1.82	0.00012	0.000506	2.98	1.16
Fst	1.59	0.000229	0.000862	9.35	7.77
IL17f	6.84	0.000231	0.000862	8.15	1.31
Casp3	1.46	0.000244	0.000869	7.2	5.74
Yes1	1.49	0.000272	0.000929	2.35	0.86
Wfikkn2	1.33	0.000293	0.000962	6.6	5.27
Ca13	1.85	0.000356	0.00112	3.34	1.49
IL1b	1.96	0.00043	0.00131	4.1	2.14
Dll1	1.58	0.00048	0.00141	5.78	4.2
Qdpr	2.21	0.000514	0.00141	5.24	3.03
Ntf3	1.44	0.000515	0.00141	3.3	1.86
Prdx5	3.95	0.000583	0.00154	5.26	1.3
Fstl3	1.44	0.000653	0.00167	7.38	5.94
Fas	2.34	0.000898	0.00216	6.47	4.13
IL1a	2.2	0.000962	0.00225	7.7	5.5
Tnni3	3.42	0.00102	0.00229	4.92	1.5
Gfra1	1.32	0.00118	0.00255	4.3	2.98
Tnfrsf12a	3.17	0.00123	0.00259	6.18	3.01
Eda2r	2.41	0.00146	0.003	8.85	6.44
Tnfrsf11b	1.69	0.00203	0.00407	7.55	5.87
Clmp	1.49	0.00209	0.00409	8.11	6.62
IL17a	4.27	0.00421	0.00802	6.82	2.55
Mia	1.37	0.00461	0.00841	6.35	4.99
Itgb6	1.31	0.00461	0.00841	3.34	2.02
Map2k6	1.18	0.00624	0.011	5.87	4.69
Apbb1ip	1.44	0.00876	0.0133	4.07	2.63
Acvrl1	1.52	0.0101	0.0147	6.11	4.58

Volvulus 24 h compared to control:

Protein	Log2FC	P-Value	FDR	Mean in group Volvulus 24h	Mean in group control
Dctn2	1.3	0.0000797	0.00313	2.46	1.16
Parp1	1.88	0.000109	0.00313	5.29	3.41
Prdx5	2.01	0.000114	0.00313	3.32	1.3
IL23r	-1.53	0.000267	0.00547	4.39	5.93
Tgfa	-1.33	0.000379	0.00621	5.55	6.89
Cyr61	-2.95	0.000864	0.0107	3.61	6.55
Tnni3	2.69	0.00249	0.017	4.19	1.5
IL1b	1.72	0.00342	0.0187	3.86	2.14
Pdgfb	-2.1	0.00892	0.0404	5.47	7.57
Ccl2	1.25	0.00937	0.0404	11.33	10.08
IL1a	-1.5	0.0104	0.0414	4	5.5

Sepsis models displayed differential expression of plasma proteins when compared with the controls *at* 48 h:

CLP 48 h compared to control:

Protein	Log2FC	P-Value	FDR	Mean in group CLP48h	Mean in group control
Tnni3	5.44	0.00000537	0.00044	6.94	1.5
Tpp1	1.91	0.0000159	0.000653	8.37	6.46
IL17f	2.95	0.0000393	0.00108	4.26	1.31
Matn2	1.96	0.0000991	0.00196	7.01	5.05
Itgb6	1.24	0.000133	0.00196	3.27	2.02
Tnfrsf11b	1.77	0.000144	0.00196	7.63	5.87
Dctn2	1.32	0.00022	0.00258	2.49	1.16
Tnfsf12	2.03	0.000299	0.00286	6.52	4.48
Eno2	1.25	0.000314	0.00286	4.85	3.6
Sez6l2	1.72	0.000382	0.00287	6.03	4.31
Cxcl1	4.14	0.000403	0.00287	12.95	8.81
Wfikkn2	1.39	0.000449	0.00287	6.66	5.27
Ntf3	2.66	0.000479	0.00287	4.53	1.86
Ccl20	3.42	0.000491	0.00287	11.38	7.96
Adam23	1.27	0.000586	0.00313	2.62	1.35
IL23r	-1.43	0.000611	0.00313	4.49	5.93
Epo	3.38	0.000742	0.00353	6.89	3.52
IL1b	2.05	0.000774	0.00353	4.19	2.14
Tgfbr3	1.15	0.000901	0.00389	5.27	4.12
IL6	4.98	0.00177	0.00655	6.75	1.77
Ccl2	2.83	0.00179	0.00655	12.91	10.08
Prdx5	2.63	0.00184	0.00655	3.93	1.3
Vegfd	1.02	0.00237	0.00779	4.93	3.91
Ccl3	4.01	0.00247	0.00779	7.03	3.02
IL17a	1.78	0.00263	0.00799	4.34	2.55
Fst	1.61	0.00384	0.0112	9.38	7.77
Cyr61	-1.72	0.00463	0.0122	4.84	6.55
Csf2	2.51	0.0055	0.0141	3.45	0.94
Tgfa	-1.23	0.00587	0.0146	5.66	6.89
Parp1	1.53	0.00674	0.0163	4.93	3.41
Fas	1.03	0.00961	0.0219	5.15	4.13
IL10	2.99	0.025	0.0489	4.93	1.94

LPS 48 h compared to control:

Protein	Log2FC	P-Value	FDR	Mean in group LPS 48h	Mean in group control
Ccl3	6.45	1.01E-11	8.31E-10	9.48	3.02
IL10	4.85	2.58E-10	1.06E-08	6.8	1.94
Ccl2	4.37	9.46E-10	2.59E-08	14.45	10.08
Nadk	2.77	0.00000012	0.00000247	8.89	6.12
Tnfrsf11b	2.52	0.000000216	0.00000355	8.38	5.87
Pla2g4a	1.71	0.000000516	0.00000705	6.47	4.76
Ccl5	4.21	0.000000975	0.0000101	5.26	1.04
Parp1	3.36	0.00000103	0.0000101	6.77	3.41
Riox2	1.84	0.00000111	0.0000101	3.91	2.07
Ccl20	3.73	0.00000306	0.0000251	11.69	7.96
Ntf3	2.21	0.00000362	0.000027	4.07	1.86
Matn2	1.73	0.00000581	0.0000397	6.78	5.05
IL6	2.14	0.00000861	0.0000491	3.91	1.77
Wfikkn2	1.51	0.0000089	0.0000491	6.78	5.27
Csf2	2.69	0.00000897	0.0000491	3.63	0.94
IL1b	2.15	0.0000103	0.0000529	4.29	2.14
Tnni3	3.38	0.0000121	0.0000585	4.88	1.5
Tnfsf12	1.8	0.0000172	0.0000743	6.28	4.48
Prdx5	2.61	0.0000178	0.0000743	3.91	1.3
Cyr61	-2.22	0.0000181	0.0000743	4.33	6.55
Adam23	1.21	0.0000346	0.00013	2.56	1.35
Fas	1.24	0.000035	0.00013	5.37	4.13
Cxcl1	3.49	0.0000492	0.000175	12.3	8.81
Dctn2	1.39	0.0000963	0.000321	2.56	1.16
Eda2r	1.43	0.0000978	0.000321	7.86	6.44
Itgb6	1.37	0.000115	0.000364	3.39	2.02
Axin1	1.02	0.000125	0.000379	4.04	3.02
Tnfrsf12a	1.68	0.000279	0.000788	4.69	3.01
Clmp	1.11	0.000292	0.000798	7.74	6.62
Cxcl9	2.93	0.000468	0.00116	8.24	5.31
IL17f	3.24	0.000512	0.00124	4.55	1.31
Pdgfb	-2.23	0.000691	0.00157	5.34	7.57
Fst	1.46	0.000967	0.00203	9.23	7.77
Ca13	1.31	0.00184	0.00343	2.8	1.49
Crim1	1.06	0.00236	0.00421	2.69	1.64
Epo	2.37	0.00292	0.00489	5.89	3.52
Ahr	1.08	0.00313	0.00513	2.81	1.73
Apbb1ip	1.24	0.00336	0.0054	3.87	2.63
IL1a	1.72	0.00595	0.00912	7.22	5.5

Volvulus 48 h compared to control:

Protein	Log2FC	P-Value	FDR	Mean in group Volvulus 48h	Mean in group control
23r	-1.74	0.000139	0.00524	4.18	5.93
IL1b	2.3	0.000196	0.00524	4.44	2.14
Epo	3.27	0.000289	0.00524	6.79	3.52
Tgfa	-1.47	0.000931	0.0109	5.41	6.89
Prdx5	1.79	0.00213	0.0205	3.09	1.3
Cyr61	-1.47	0.00271	0.0223	5.08	6.55
IL6	1.01	0.00506	0.0323	2.78	1.77
Pdgfb	-2.23	0.00511	0.0323	5.34	7.57
Gcg	-1.81	0.00596	0.0326	1.74	3.55
Ccl2	1.04	0.00927	0.0447	11.13	10.08
Parp1	1.21	0.0108	0.0492	4.62	3.41

Supplement III. In CLP and volvulus the differential plasma protein expression is stable in the interval between 24 and 48 h after intervention.

(A) In CLP only 1 protein was upregulated, (B) in volvulus the protein expression was stable and (C) in the LPS model 3 proteins were upregulated and 10 were downregulated. Expression was considered to differ significantly when FDR ≤ 0.05 and |log2FC| ≥ 1. The differentially expressed plasma proteins are listed in supplement III.

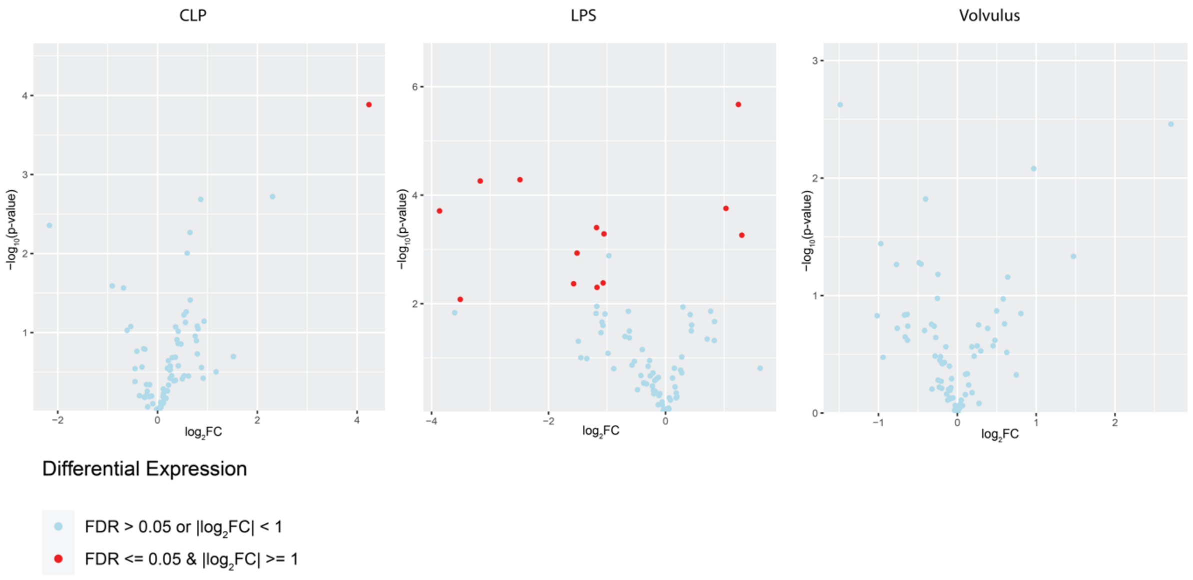

Supplement IV:

In CLP and volvulus, the differential plasma protein expression is stable in the interval between 24 h and 48 h after intervention. Differential expression of only very view proteins between the two timepoints is observed:

CLP 24 h compared to CLP 48 h:

Protein	Log2FC	P-Value	FDR	Mean in group CLP48h	Mean in group CLP24h
Epo	4.24	0.000131	0.0107	6.89	2.66

LPS 24 h compared to LPS 48 h:

Protein	Log2FC	P-Value	FDR	Mean in group LPS48h	Mean in group LPS24h
Adam23	1.25	0.00000213	0.000175	2.56	1.31
IL6	-2.49	0.0000519	0.0015	3.91	6.4
Ccl5	-3.17	0.0000549	0.0015	5.26	8.42
S100a4	1.03	0.000175	0.00321	5.16	4.13
Cxcl9	-3.86	0.000195	0.00321	8.24	12.1
Matn2	-1.18	0.000396	0.00542	6.78	7.96
Clstn2	-1.05	0.000518	0.0056	2.75	3.8
Ccl3	1.3	0.000546	0.0056	9.48	8.17
Csf2	-1.51	0.00117	0.0106	3.63	5.15
Fstl3	-1.07	0.00416	0.0293	6.31	7.38
Qdpr	-1.57	0.00429	0.0293	3.67	5.24
Mia	-1.17	0.00501	0.0316	5.18	6.35
IL17a	-3.51	0.00835	0.0489	3.31	6.82

Volvulus 24 h compared to 48 h volvulus:

Protein	Log2FC	P-Value	FDR	Mean in group Volvulus 48h	Mean in group Volvulus 48h
none	none	none	none	none	none

Supplement V:

	**Ccl3**	**Il1b**	**Lpl**	**Wisp1**	**Pla2g4a**
**CLP24**	3.96	2.01	n.s.	n.s.	n.s.
**CLP48**	4.91	2.05	n.s.	n.s.	n.s.
**LPS24**	5.15	1.96	n.s.	n.s.	1.92
**LPS48**	6.45	2.15	n.s.	n.s.	1.71
**Volvulus 24**	n.s.	1.72	n.s.	n.s.	n.s.
**Volvulus 48**	n.s.	n.s.	n.s.	n.s.	n.s.

Differential expression of proinflammatory proteins in each of the three models at the timepoints 24h and 48h, expressed as Log2FC. Positive value indicated upregulation of protein expression, negative values indicate downregulation of proteins.

Supplement VI:

	IL10	Ghrl	Hgf
**CLP24**	1.81	n.s.	n.s.
**CLP48**	2.99	n.s.	1.07
**LPS24**	4.67	n.s.	n.s.
**LPS48**	4.85	n.s.	n.s.
**Volvulus 24**	n.s.	n.s.	n.s.
**Volvulus 48**	n.s.	n.s.	n.s.

Differential expression of anti-inflammatory proteins in each of the three models at the timepoints 24h and 48h, expressed as Log2FC. Positive value indicated upregulation of protein expression, negative values indicate downregulation of proteins. Significance was defined when FDR ≤ 0.05 and |log2FC| ≥ 1 was met.

Supplement VII:

Exemplary illustration of the Midgut Volvulus procedure. (A) Normal position, (B) Rotation: After performing median laparotomy, the small intestine is exposed and twisted by 720°. The axis of rotation is selected only just that the intestine turns livid and that signs of congestion become slightly apparent.

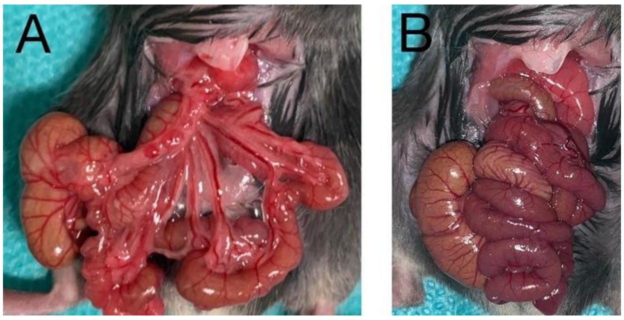

Supplement: Supplementary file 1 [file cells-12-00366-s001.zip › cells-2050769-supplementary.pdf]
